# Integration of stereotactic radiotherapy in the treatment of metastatic colorectal cancer patients: a real practice study with long-term outcome and prognostic factors

**DOI:** 10.18632/oncotarget.25834

**Published:** 2018-10-16

**Authors:** Alessandro Ottaiano, Valerio Scotti, Chiara De Divitiis, Monica Capozzi, Carmen Romano, Antonino Cassata, Rossana Casaretti, Lucrezia Silvestro, Anna Nappi, Valeria Vicario, Alfonso De Stefano, Salvatore Tafuto, Massimiliano Berretta, Guglielmo Nasti, Antonio Avallone

**Affiliations:** ^1^ Department of Abdominal Oncology, SSD–Innovative Therapies for Abdominal Metastases, Istituto Nazionale Tumori di Napoli G. Pascale IRCCS, National Cancer Institute, 80131, Naples, Italy; ^2^ San Rossore Clinic, Viale delle Cascine, 56122, Pisa, Italy; ^3^ Department of Abdominal Oncology, Experimental Clinical Oncology, Istituto Nazionale Tumori di Napoli G. Pascale IRCCS, National Cancer Institute, 80131, Naples, Italy; ^4^ Department of Medical Oncology, CRO Aviano, National Cancer Institute, 33081, Aviano, Italy

**Keywords:** colorectal cancer, radiation therapy, chemotherapy, metastatic colorectal cancer

## Abstract

**Background:**

There are very few clinical or prognostic studies on the role of SRT (Stereotactic Radiation Therapy) in the continuum of care of metastatic colorectal cancer (mCRC) patients.

**Patients and methods:**

Patients affected by oligo-mCRC were treated with SRT before or after front-line standard treatments. SRT was delivered according to a risk-adapted protocol. Total body CT (Computed Tomography) scan was done before therapy and every three months thereafter. The radiologic responses to therapy were evaluated by RECIST (Response Evaluation *Criteria* In Solid Tumors). FDG-PET (FluoroDeoxyGlucose - Positron Emission Tomography) was done before and after SRT; metabolic responses were evaluated by using the EORTC (European Organization for Research and Treatment of Cancer) criteria. The Kaplan-Meier product limit method was applied to graph Overall Survival (OS) and Progression-Free Survival (PFS).

**Results:**

Forty-seven patients were included. Twenty-one patients had disease limited to lungs, 9 to lung and liver, 7 only to liver, 10 to multiple sites. The median prescription SRT dose was 60 Gy per organ in 3 fractions (median biological effective dose of 180 Gy). The reduction of delta SUVmax (maximum *Standardized Uptake* Value) correlated with the local control (p<0.001) and two-years survival (p=0.003). At univariate analysis, localization of primary tumor, site of metastases, KRAS (Kirsten RAt Sarcoma) oncogene mutational status, response to first-line chemotherapy, response to SRT and number of treated lesions predicted both PFS and OS.

**Discussion:**

This real practice experience suggests that further studies are needed to analyze the promising role of SRT in the multidisciplinary management of mCRC.

## INTRODUCTION

Colorectal cancer is the third most common cancer worldwide. Despite progresses in the screening allowing for early diagnosis and definitive surgical removing of the localized tumors, about 30% of patients presents with advanced disease involving liver in more than 50% of cases [1]. Other organs frequently targeted by metastatic colorectal cancer (mCRC) are lungs and lymphnodes. The mainstay of pluri-mCRC treatment is chemotherapy (fluoropyrimidines, irinotecan, oxaliplatin) in association with new biologic drugs (bevacizumab, aflibercept, cetuximab and panitumumab); these drugs have improved survival reaching a median survival of about 30 months in selected patients [2].

In last years, the management of advanced disease has been enriched of integrated strategies including SRT (Stereotactic Radiation Therapy). The administration of SRT demonstrated, particularly in oligo-metastatic disease, to be a safe and effective option [3]. A definition of oligo-metastatic disease is the cancer spreading beyond the primary tumor involving one to three lesions per organ with a cumulative maximum tumor diameter per organ smaller than 7 cm [4]. However, oligo-metastatic disease is a dynamic and biologic state of cancer rather than the simple number and/or volume of the lesions so that its definition is difficult to approach and depends also on the instrumental tools used for detection [5]. Further molecular and biological markers identifying oligo-metastatic disease are urgently needed.

Many factors prompt the integration of SRT in the multidisciplinary therapeutic management of mCRC: i) the radiosensitivity of colorectal cancer, ii) the reduced toxicity (sparing of healthy tissue, high and hypofractionated irradiation doses) with the intriguing possibility of concomitant therapies, iii) the potential to induce immune system modulation with regression of tumor deposits in non-irradiated regions (abscopal effect), iv) the increasing availability of the technique. Several studies suggested a significant survival increase versus historical controls in patients bearing lung metastases with two-years survivals ranging from 67.7 % to 77.0% and medians surpassing 30 months [6–8]. The most important prognostic factors were the number and volume of metastatic lesions. However, there are neither prospective nor randomized studies comparing SRT versus standard therapies.

Here we report the outcome of 47 patients treated from 2007 to 2012 with SRT for mCRC; the majority of patients had oligo-metastatic disease. In half of the cases the disease involved multiple sites (lungs, liver and/or abdominal lymphnodes).

## RESULTS

### Patients, disease and treatment characteristics

Patients, diseases and treatment characteristics are shown in Table 1. Median age of patients was 67 years (range: 45-81). Twenty-seven patients were male, 20 female. Most of patients had a PS ECOG 0, 12 PS ECOG 1, 4 PS ECOG 2. The most common primary site was the left colon (15 patients), followed by right colon (10) and sigma (8). Twenty-one patients had disease limited to lungs, 9 to lung and liver, 7 only to liver; ten patients had also metastases to abdominal lymphnodes (>15 mm at TC scan and SUV>3 at PET scan). Twenty-nine tumors had wild-type KRAS, 18 mutated. Twenty-nine patients underwent to front-line SRT; in 18 patients SRT was performed after a first-line chemotherapy. Thirty-five patients were treated with more than one line of SRT [at the same sites (re-irradiation) or at different sites].

**Table 1:** Characteristics of patients and disease

Characteristics	No.
**Age, years**	
Median	67
Range	45-81
**Gender**	
Male	27
Female	20
**Performance Status**	
0	31
1	12
2	4
**Site of primary tumor**	
Rectum	5
Sigma	8
Left colon	15
Trasversum	6
Right colon	10
Cecum	3
**Site of metastases**	
Only lung	21
Only liver	7
Lung and liver	9
Lung and abdominal lymphnodes	7
Liver and abdominal lymphnodes	3
**KRAS mutational status**	
Wilde-type	29
Mutated	18
**No. of systemic treatments before first-line SRT**	
0	29
1	18
**No. of SRT lines**	
1	12
2	20
3	13
≥4	2

### SRT according to metastatic sites

In Table 2 we show the detailed extent of disease *per* patient at first-line SRT treatment. Most of patients had technically resectable oligo-metastatic disease (29 patients); however, they refused surgery (16 patients, two of them relapsed after previous lung metastasectomies) or had comorbidities (13 patients) (renal failure and/or cardiac diseases and/or hepatic diseases, etc.) contraindicating metastasectomies or aggressive front-line chemotherapies (4 out of these patients did not receive chemotherapy, 2 received capecitabine and bevacizumab at reduced doses, 7 capecitabine and oxaliplatin at reduced doses). The SRT treatment was well tolerated; only three patients experienced persistent cough for 20 to 40 days after the last irradiation.

**Table 2:** Distribution of metastatic lesions and reasons for performing SRT treatment

Patient identification number	Oligometastatic disease *ab initio*	Reasons for first-line SRT	Distribution of lesions at first SRT	Total number of lesions
**1**	No	NA	3 lung, 2 liver	5
**2**	Yes	Refusal of metastasectomies	3 lung	3
**3**	Yes	Refusal of metastasectomies	1 lung, 2 liver^B^	3
**4**	Yes	Comorbidities	3 lung, 2 abdominal LN	5
**5**	Yes	Refusal of metastasectomies	2 liver	2
**6**	No	NA	2 lung	2
**7**	No	NA	4 lung	4
**8**	Yes	Refusal of metastasectomies	2 lung	2
**9**	Yes	Refusal of metastasectomies	3 liver	3
**10**	No	NA	2 lung, 2 liver	4
**11**	Yes	Refusal of metastasectomies	3 lung	3
**12**	No	NA	3 lung, 2 abdominal LN	5
**13**	Yes	Comorbidities	6 lung	6
**14**	Yes	Refusal of metastasectomies	2 lung, 1 abdominal LN	3
**15**	No	NA	2 liver, 2 abdominal LN	4
**16**	Yes	Refusal of metastasectomies	3 liver^B^	3
**17**	Yes	Refusal of metastasectomies	1 lung	1
**18**	No	NA	2 lung	2
**19**	Yes	Comorbidities	3 liver, 2 abdominal LN	5
**20**	Yes	Comorbidities	4 liver^B^	4
**21**	Yes	Refusal of metastasectomies	2 lung	2
**22**	No	NA	3 lung, 1 abdominal LN	4
**23**	No	NA	5 lung	5
**24**	No	NA	4 lung	4
**25**	Yes	Comorbidities	2 lung, 1 abdominal LN	3
**26**	Yes	Comorbidities	3 liver, 2 abdominal LN	5
**27**	Yes	Refusal of metastasectomies	2 lung	2
**28**	No	NA	4 lung, 3 liver	7
**29**	Yes	Comorbidities	2 lung, 1 liver	3
**30**	Yes	Refusal of metastasectomies	3 liver^B^	3
**31**	Yes	Comorbidities	5 liver	5
**32**	Yes	Comorbidities	4 lung, 2 liver	6
**33**	No	NA	3 lung	3
**34**	Yes	Refusal of metastasectomies	1 lung, 1 liver	2
**35**	Yes	Comorbidities	6 lung	6
**36**	Yes	Refusal of metastasectomies	3 lung	3
**37**	No	NA	2 lung	2
**38**	No	NA	5 lung, 2 abdominal LN^B^	7
**39**	Yes	Refusal of metastasectomies	2 lung	2
**40**	Yes	Comorbidities	3 lung, 1 abdominal LN	4
**41**	No	NA	5 lung	5
**42**	Yes	Refusal of metastasectomies	1 lung	1
**43**	Yes	Comorbidities	3 lung, 3 liver	6
**44**	No	NA	3 lung	3
**45**	No	NA	2 lung	2
**46**	Yes	Comorbidities	3 liver^B^	3
**47**	No	NA	4 lung, 3 liver	7

NA: Not Applicable, in these cases SRT was performed after chemotherapy for poly-metastatic disease on “residual” masses.

^B^These patients experienced progressive disease at first-line chemotherapy.

### Systemic treatments and toxicities

Treatments are depicted in details in Table 3. Forty-three patients received a first-line chemotherapy; the most common schedule was the association between fluoropyrimidines (capecitabine or fluorouracil), oxaliplatin and bevacizumab (Capox or Folfox plus Bevacizumab). Median duration of treatments was 7.3 months. Four patient did not undergo to chemotherapy because of comorbidities and age. The use of fluoropyrimidines, irinotecan and anti-EGFR (cetuximab or panitumumab) was predominant in second-line therapies. Monotherapies with anti-EGFR agents and fluoropyrimidines and mitomycin-c association were more frequent in third-line treatments. Twenty-one patients were re-treated with previous therapies (if the progression-free interval was more than 6 months and there were not previous grade 3/4 toxic events). There were no grade 4 toxicities or toxic deaths. The most common G3 adverse events were diarrhea (13/47 patients), neutropenia (11/47 patients) and cutaneous rush (6/47).

**Table 3:** Chemotherapeutic regimens

First-line schedules	No. of patients
Folfox or Capox	9
Folfox or Capox + Bevacizumab	23
Folfiri or CapIri	1
Folfiri or CapIri + Bevacizumab	2
Folfiri or CapIri + anti-EGFR	6
Irinotecan + anti-EGFR	0
Fluoropyrimidines monotherapy	0
Fluoropyrimidines + Bevacizumab	2
Anti-EGFR monotherapy	0
Fluoropyrimidines + Mytomicin-C	0
**Second-line schedules**	
Folfox or Capox	7
Folfox or Capox + Bevacizumab	0
Folfiri or CapIri	8
Folfiri or CapIri + Bevacizumab	2
Folfiri or CapIri + anti-EGFR	15
Irinotecan + anti-EGFR	4
Fluoropyrimidines monotherapy	3
Fluoropyrimidines + Bevacizumab	0
Anti-EGFR monotherapy	1
Fluoropyrimidines + Mytomicin-C	0
**Third line schedules**	
Folfox or Capox	1
Folfox or Capox + Bevacizumab	0
Folfiri or CapIri	3
Folfiri or CapIri + Bevacizumab	0
Folfiri or CapIri + anti-EGFR	0
Irinotecan + anti-EGFR	3
Fluoropyrimidines monotherapy	4
Fluoropyrimidines + Bevacizumab	0
Anti-EGFR monotherapy	6
Fluoropyrimidines + Mytomicin-C	9
**Re-challenges at any lines of therapy**	21

### Associations between SRT and clinical variables

**Figure 1:**
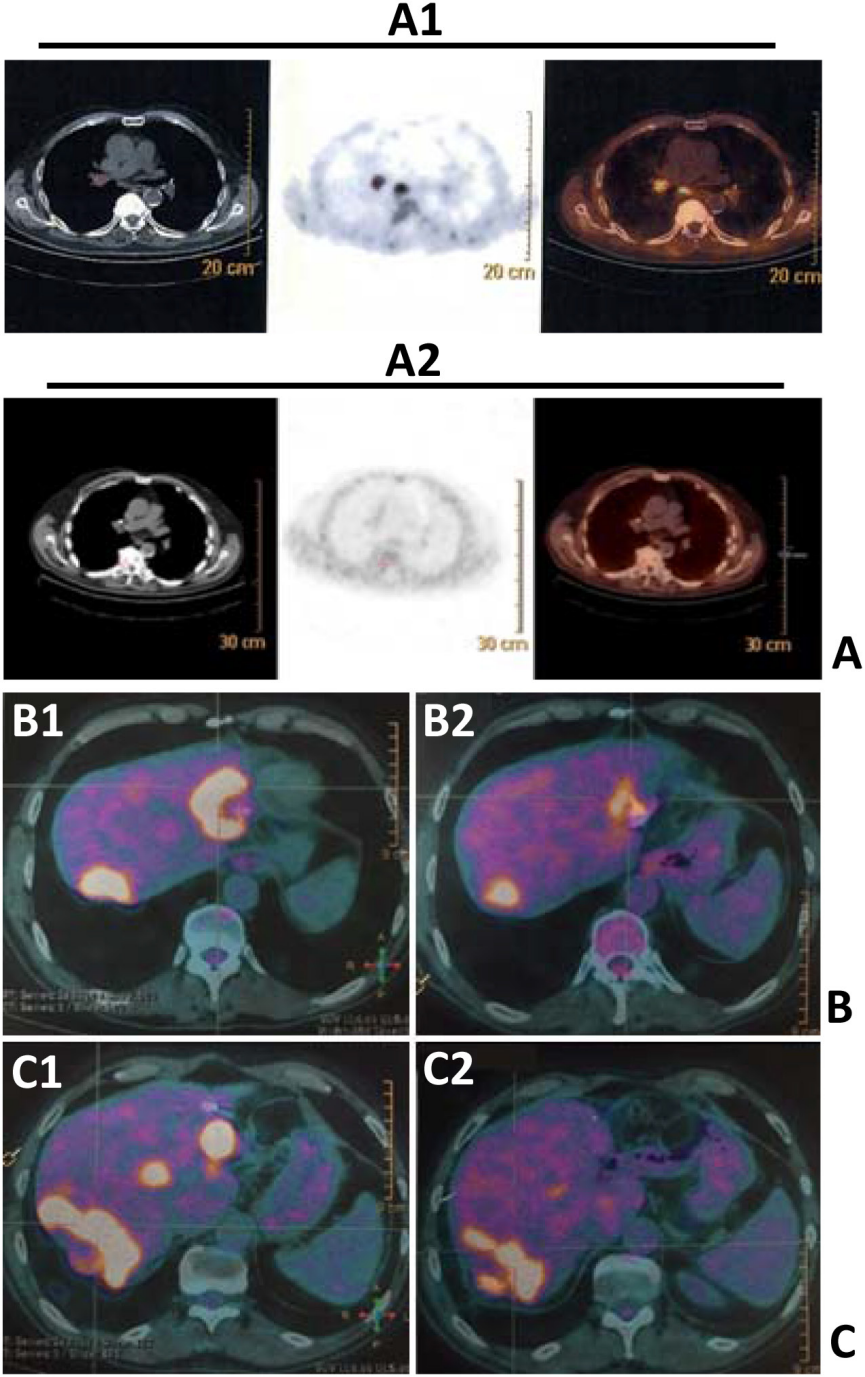
FDG-PET scans showing FDG uptakes before (A1, B1 and C1) and after SRT (A2, B2 and C2) in lungs (A1 vs A2) and liver metastases (B1 vs B2; C1 vs C2) of three patients (panels **A, B, C**).

In our series, all patients performed FDG-PET before and after the first administration of SRT. Figure 1 shows FDG-PET results of SRT in three patients. The median prescription dose was 60 Gy per organ in 3 fractions (median biological effective dose of 180 Gy). The reduction of deltaSUV_max_ correlated with the local control of disease (time-to-progression after SRT at the irradiated sites, p<0.001) and survival at two years (p=0.003). Interestingly, although not significant (p=0.0512) for the low numbers included, patients who received a first-line chemotherapy including bevacizumab (anti-VEGF) before SRT were more prone to respond to SRT compared to patients treated with chemotherapy only. There were no significant correlations between delta SUVmax (maximum *Standardized Uptake* Value)and timing of chemotherapy (before or after SRT), response to first-line treatment, and sites of disease (Table 4). Given the importance of the metastatic site in the decision-making and planning of SRT, with an exploratory and descriptive aim, potential correlations between sites of disease and FDG-PET responses after SRT were studied (Table 5a and 5b). The patients were divided into two groups: i) upfront SRT *vs* ii) SRT after front-line chemotherapy. In fact, in the second group, responses to SRT could be influenced by the previous exposition of neoplastic cells to chemotherapy. There were no significant differences in terms of FDG-PET responses to SRT between different sites into the two groups; however, most of CMR and PMR according to EORTC criteria were observed in lung-limited disease patients

**Table 4:** Correlations between ΔSUV_max_ and clinical variables

	No. of patients	DSUV_max_ median of the treated lesions (standard deviation)	*P*
**Local control (months)**^A^			
<6	10	42 (28)	
6-12	12	62 (18)	
>12	25	78 (23)	<0.001
**Chemotherapy before SRT**			
No	29	64 (16)	
Yes	18	68 (22)	0.293
**Response to first-line chemotherapy**			
RC, RP, SD	37	69 (26)	
PD	6	62 (19)	0.348
**Anti-VEGF therapy before SRT**			
Yes	11	73 (15)	
No	5	56 (20)	0.0512
**Overall survival**			
≤2 years	9	46 (17)	
>2 years	38	75 (22)	0.003
**Sites of disease**			
Lung	21	67 (16)	
Liver	7	58 (28)	
Lung and liver	9	71 (15)	
Presence of LN metastases	10	77 (20)	0.091
**KRAS mutational status**			
Wilde Type	29	69 (14)	
Mutated	18	61 (19)	0.462

A: from the date of the first SRT application to the evidence of progression of the irradiate site.

**Table 5a:** FDG-PET responses in patients receiving upfront SRT (29 patients)

		FDG-PET response			
		CMR	PMR	SMD	PMD
**Sites of disease**					
Lung	11	1	7	2	1
Liver	7	2	4	0	1
Lung and liver	5	0	2	1	2
Presence of LN metastases	6	1	1	2	1

**Table 5b:** FDG-PET responses in patients receiving SRT after first-line line chemotherapy (18 patients)

		FDG-PET response			
		CMR	PMR	SMD	PMD
**Sites of disease**					
Lung	10	2	6	2	0
Liver	0	0	0	0	0
Lung and liver	4	0	2	1	1
Presence of LN metastases	4	0	2	0	2

CMR: complete metabolic response; PMR: partial metabolic response; SMD: stable metabolic disease; PMD: progressive metabolic disease.

^*^All PMDs were attributable to new lesions distant form the irradiated sites.

### Clinical and pathological prognostic factors in patients treated with SRT

**Figure 2:**
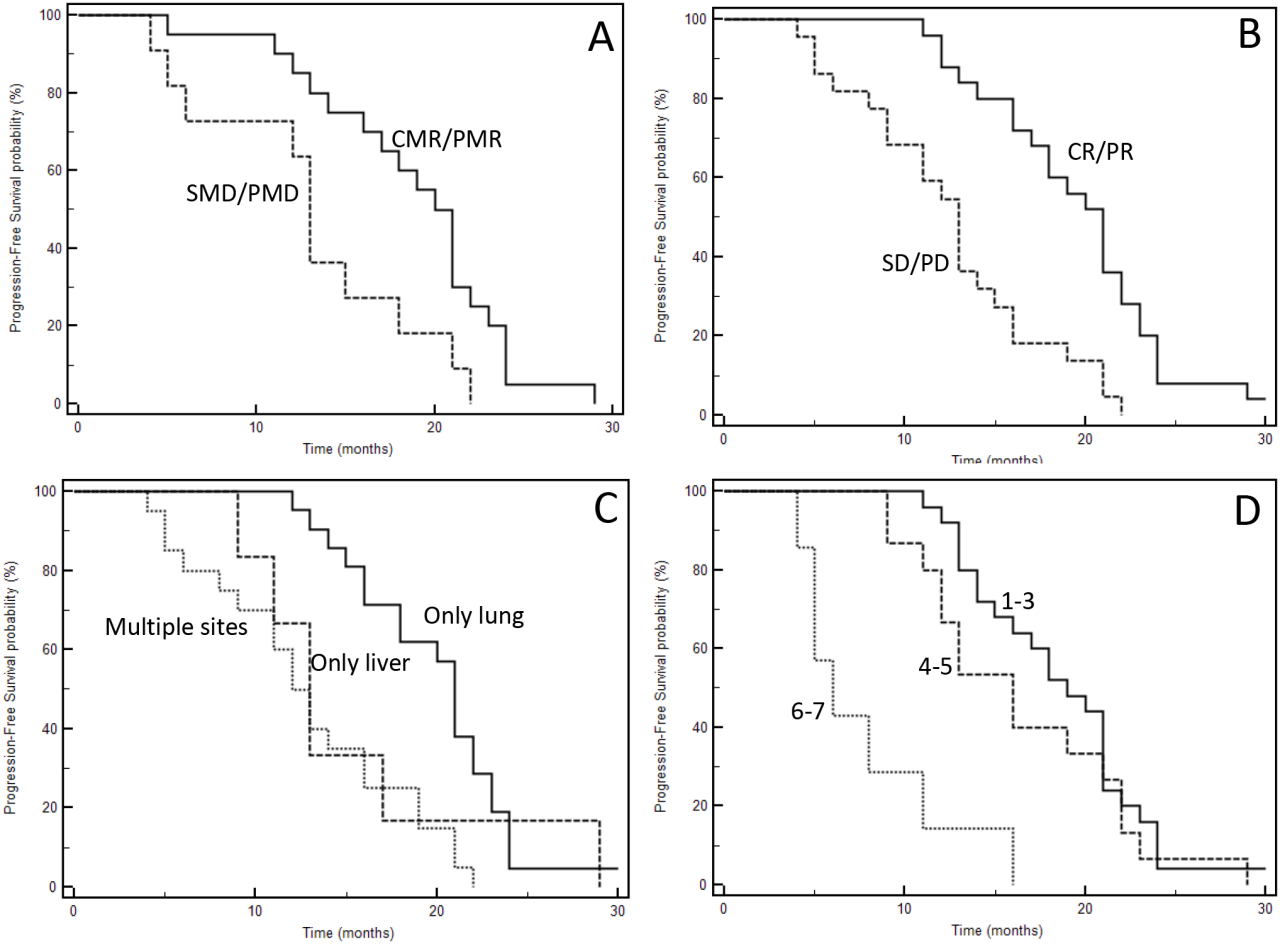
Kaplan-Meyer progression-free survival curves according to response to therapies (**A**: SRT; **B**: chemotherapy), and extent of disease (**C**: type of involved organ; **D**: total number of metastases). See Table 6 for P at log-rank test.

**Figure 3:**
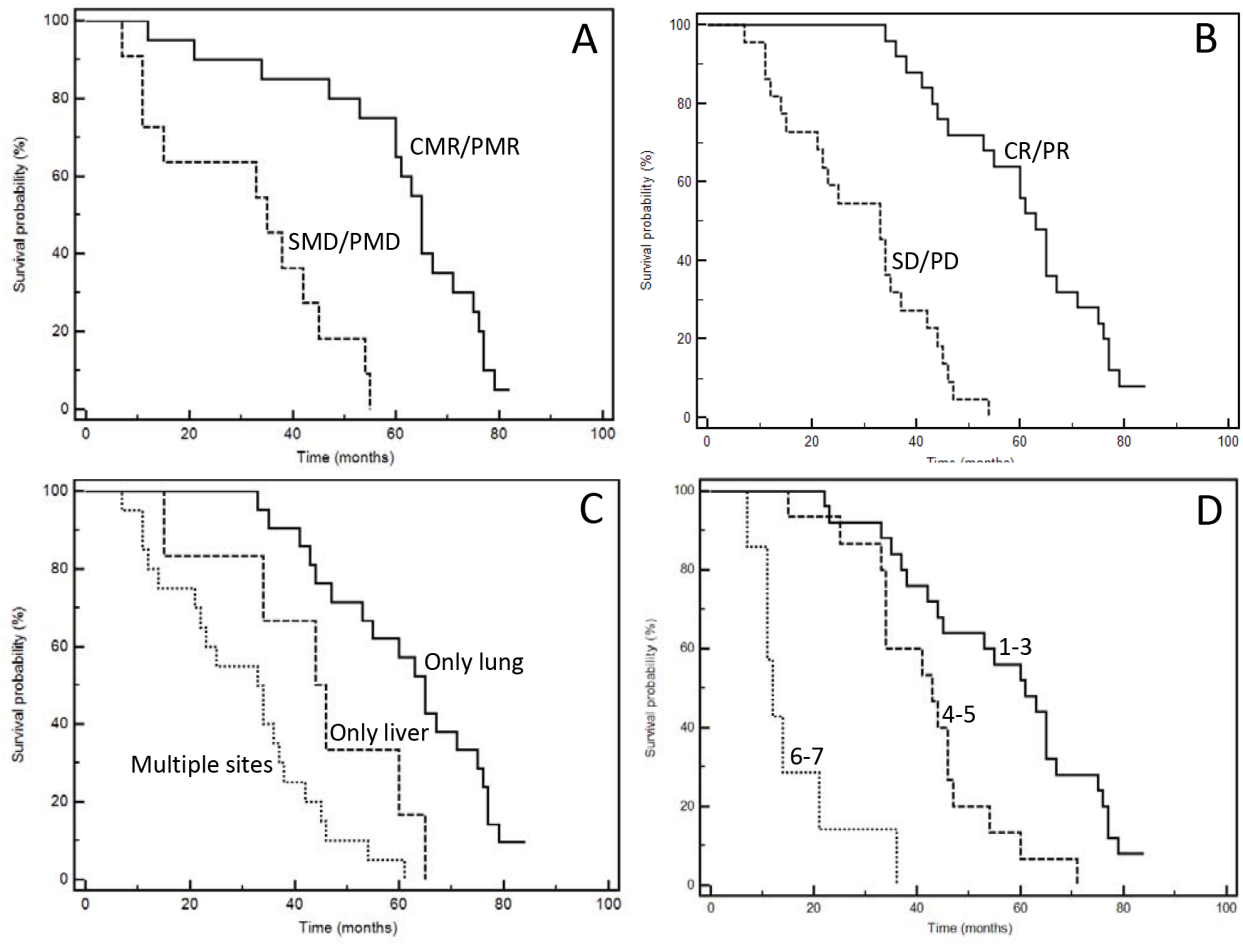
Kaplan-Meyer survival curves according to response to therapies (**A**: SRT; **B**: chemotherapy), and extent of disease (**C**: type of involved organ; **D**: total number of metastases). See Table 7 for P at log-rank test.

One of the most important characteristic of our series is the mature follow-up (median follow-up: 48.8 months); forty-five events were registered, only two patients are still alive at the time of last follow-up (May, 16 2017). The median PFS of the entire series was 16 months; median OS 44.0 months. Figure 2 and 3 show Kaplan-Meyer PFS and OS curves according to response to systemic therapy or SRT and extent of the disease. At univariate analysis, localization of primary tumor, site of metastases, KRAS mutational status, response to first-line chemotherapy, response to upfront SRT evaluated with FDG-PET and number of treated lesions had a positive prognostic power (Table 6 and 7).

**Table 6:** Univariate analysis of progression-free survival according to specific clinical, molecular and anatomical variables

Variable	Events/Patients	Median PFS^1^ (months)	HR^2^	95% CI^3^	*P* at univariate
Age (≤70 vs >70 years)	15/16 vs 31/31	16 vs 16	1.29	0.71-2.34	0.37
Gender (male vs female)	26/27 vs 20/20	14 vs 20	0.86	0.48-1.53	0.57
Localization of primary tumor (right vs left colon)	24/24 vs 22/23	13 vs 19	1.70	0.94-3.09	0.0445
Site of metastases (only lung vs only liver vs multiple sites)	20/21 vs 6/6 vs 20/20	21 vs 13 vs 12	0.36	0.18-0.73	0.0014
KRAS mutational status (mutated vs wild-type)	18/18 vs 28/29	12 vs 18	2.02	1.02-3.97	0.0093
Response to first-line chemotherapy (RC/RP vs SD/ PD)	25/26 vs 21/21	21 vs 13	0.35	0.18-0.69	<0.0001
Response to first-line SRT (CMR/PMR vs SMD/PMD)	20/20 vs 11/11	20 vs 13	0.43	0.17-1.05	0.0199
Number of lesions (1-3 vs 4-5 vs 6-7)	24/25 vs 15/15 vs 7/7	19 vs 16 vs 6	0.14	0.02-0.85	<0.0001

PFS^1^= Progression-Free Survival; HR^2^ = Hazard Ratio; CI^3^ = Confidence Intervals.

**Table 7:** Univariate analysis of overall survival according to specific clinical, molecular and anatomical variables

Variable	Events/Patients	Median OS^1^ (months)	HR^2^	95% CI^3^	*P* at univariate
Age (≤70 vs >70 years)	14/16 vs 31/31	41.5 vs 46	1.28	0.69-2.35	0.42
Gender (male vs female)	25/27 vs 20/20	44 vs 45	0.97	0.54-1.75	0.92
Localization of primary tumor (right vs left colon)	19/19 vs 26/28	38 vs 60	2.03	1.11-4.68	0.0008
Site of metastases (only lung vs only liver vs multiple sites)	19/21 vs 6/6 vs 20/20	65 vs 45 vs 33	0.28	0.15-0.55	<0.0001
KRAS mutational status (mutated vs wild-type)	18/18 vs 27/29	34 vs 55	2.47	1.21-5.05	0.0012
Response to first-line chemotherapy (RC/RP vs SD/ PD)	24/26 vs 21/21	63 vs 33	0.23	0.10-0.49	<0.0001
Response to first-line SRT (CMR/PMR vs SMD/PMD)	19/20 vs 11/11	65 vs 35	0.22	0.07-0.69	<0.0001
Number of lesions (1-3 vs 4-5 vs 6-7)	23/25 vs 15/15 vs 7/7	61 vs 43 vs 12	0.23	0.02-0.77	<0.0001

OS^1^= Overall Survival; HR^2^ = Hazard Ratio; CI^3^ = Confidence Intervals.

## DISCUSSION

Patients with mCRC may present with an oligo-metastatic disease with neoplastic lesions approachable with local treatments. In last years, many studies have been published reporting results of SRT in the treatment of oligo-mCRC [9–23]. They were heterogeneous in terms of number of treated patients (from 13 [17] to 82 [20]) and median overall survivals (from 16.0 months [10] to 46.0 [21]). Median follow-up was often inferior to 35 months with the longest one reported by *Agolli et al.* of 36 months [15]. In our study we show the outcome as well as the prognostic factors of 47 consecutive patients affected by oligo-mCRC treated with first or subsequent lines of SRT from 2007 to 2012. Notably, most of patients had a local control >12 months, which is similar to previous experiences. Furthermore, the median overall survival in our series, including patients with both lung and liver involvement (9 patients) or diffusion to abdominal lymphnodes (10 patients) of 44.0 months, was particularly positive considering that the best survival previously reached with SRT (46.0 months) was reported in patients with only lung metastases [21]. Interestingly, the five-year survival of patients with lung-limited disease was 39%, this data compares well with historical surgical controls [21–24] considering also that 12 patients had ≥3 lesions and that some patients received a “depotentiated” chemotherapy course because of comorbidities or age.

The more positive outcome of our study could be attributable to the introduction of new therapies (anti-VEGF, anti-EGFR agents) in the context of a continuum of care strategy. However, many patients had comorbidities excluding the administration of standard or continuous chemotherapies. A possible speculative explanation of significantly better results can rely also on different lines of SRT (re-irradiations) reinforcing the abscopal effect of radiotherapy which consists on the local induction of tumor antigens and the release of cytokines stimulating in turn the innate and adaptive immunity [25]. However, this is a perspective to verify because in the present study, no immunologic evaluations were done. In future studies, prospective evaluations of immune-regulatory cells (Tregs [regulatory T cells], MDSC [myeloid-derived suppressor cells]) [26], effector cells (NK [Natural Killer] and T lymphocytes) [27], cytokines [28] and correlations with immunescores [29, 30] (on primary and/or metastatic lesions) will be necessary to clarify the immunologic mechanisms eventually underling the clinical outcomes. However, to this regard, many clinical and translational trials in advanced lung, melanoma and mCRC are now recruiting patients through protocols based on SRT and immunotherapies with different mechanisms of action (pembrolizumab, durvalumab, tremelimumab, dabrafenib, trametinib, MK-3475, etc.) (https://clinicaltrials.gov/). The intent of these studies is to take advantage from the immune-modulating properties of SRT in synergism with immunotherapeutic drugs to improve the anti-tumor effects.

Interestingly, some patients presented with poly-metastatic multi-organs disease and received a standard first-line chemotherapy as front-line therapy; in these cases, SRT was administered in “residual” disease. These patients are more similar to mCRC patients with more aggressive and widely metastatic tumors. They did not receive systemic therapy after SRT until progression. The univariate analysis showed a worse prognosis compared to patients with only lung or liver involvement; however, they had a median survival of 34 months which is superior to the survival described in last generation randomized trials [31, 32]. Lacking specific studies (ideally, chemotherapy plus SRT vs chemotherapy), this is an indirect evidence that the integration of SRT in advanced disease could ameliorate the anti-neoplastic effect of chemotherapy and contribute to the control of systemic disease.

The neoplastic lesions responded to SRT independently from i) the primary or metastatic site, ii) the administration of upfront chemotherapy, iii) chemosensitivity of the tumor (evaluated as response to a first-line chemotherapy). Interestingly, the administration of bevacizumab before irradiation was slightly associated with the metabolic response (p=0.0512). Although not significant, this could be related to the effect of bevacizumab on tumor vasculature with reduction in microvessel density producing an increase of tumor oxygenation and perfusion; these phenomena are associated with increased sensitivity to radiation therapy in tumor models [33, 34].

KRAS mutational status was not associated with response to SRT, but predicted PFS and OS at univariate analysis. The best prognostic profile in this study was represented by left sided, KRAS wild-type, lung-limited tumor. The presence of these three conditions were strongly associated with survival compared to other prognostic combinations (+33.5 months; 75.5 vs 42 months; HR: 0,30; 95% CI: 0.16-0.56; p=0.0021 at log-rank test) and with response to SRT and chemotherapy (data not shown).

One of the major limitation of our study consists on the heterogeneity of patient cohort with regard to systemic treatment protocols, radiotherapy regimens (doses and timing with chemotherapy) and Kras status that, in absence of a comparator (no SRT), makes interpretation of the findings as predominantly descriptive and exploratory. However, the increasing availability of SRT along with the shorter treatment duration, the high precision and the high sparing of surrounding normal tissues, make this technique a valid option in the treatment of oligo-mCRC; in left-sided, KRAS wild-type, lung-limited tumors it could be a valid alternative to surgery. Furthermore, our experience suggests that SRT could contribute to obtain a long-term disease control also when multiple organs are involved; prospective and larger studies are needed to confirm these data.

## MATERIALS AND METHODS

### Patient management and follow-up

Patients were treated at the Department of Abdominal Medical Oncology of the National Cancer Institute (Naples, Italy) from 2007 to 2012. Only two patients of the present cohort did not undergo to primary tumor resection because it was asymptomatic. All primary tumors were routinely characterized for KRAS (Kirsten RAt Sarcoma) oncogene mutational status. Six patients were stage III at diagnosis but they presented distant metastases three months after surgery at the first follow-up (synchronous metastases). Sequential standard treatments with chemotherapy (fluorouracil/capecitabine, irinotecan, oxaliplatin) and/or biologic therapies (bevacizumab, cetuximab, panitumumab) were administered. The choice of chemotherapy regimen was based on patient's performance status, extent of disease, comorbidities, previous treatments and individual preferences. Informed consent from each patient was sought. Total body computed tomography (CT) scan and CEA (CarcinoEmbryonic Antigen) monitoring were done every three months. The response to therapy was evaluated by RECIST (Response Evaluation *Criteria* In Solid Tumors). Patients with target metastatic lesions restaged at the Radiology Unit were considered for response evaluation. Complete response (CR) was defined as complete disappearance of all detectable evidence of disease on total body computed tomography. Partial response (PR) was defined as at least a 30% decrease in the sum of diameters of target lesions. Stable disease (SD) was defined as everything between 30% decrease and 20% growth of tumor size. Progressive disease (PD) was defined as at least a 20% increase in the sum of diameters of target lesions. Toxicity was graded with the Common Toxicity Criteria for Adverse Events (CTCAE) v3.0. Local control was defined from last day of SRT to local relapse within the irradiated site. RECIST was only used to assess response to chemotherapy.

FDG-PET (FluoroDeoxyGlucose-Positron Emission Tomography) responses were evaluated by using the European Organization for Research and Treatment of Cancer (EORTC) criteria [35, 36]. In brief, definitions of metabolic response by FDG-PET/CT included: complete metabolic response (CMR: complete resolution of all metabolically active target and non-target lesions, and no new lesions); partial metabolic response (PMR: 20% or greater decrease in SUV of target lesions with or without decrease in number/size of nontarget lesions, and no new lesions); progressive metabolic disease (PMD: one or more new lesions, 20% or greater increase in SUV of target lesions and/or unequivocal increase in FDG activity of nontarget lesions); and stable metabolic disease (SMD: not qualifying as CMR, PMR, or PMD). FDG PET responses were also evaluated with the Response Index (RI). The measurements of SUV (Standardized Uptake Value) obtained in the metastatic lesions at baseline (SUV_1_) and after 60 days from SRT treatment (SUV_2_) were compared and the change was expressed as the percentage of SUV reduction (ΔSUV = (SUV_1_−SUV_2_)/SUV_1_×100).

### SRT indications

Our policy was to propose SRT in patients with one to six lesions in the lungs, one to three lesions into the liver, with a cumulative maximum tumor diameter *per* organ smaller than 7 cm or in patients who refused surgery or had comorbidities contraindicating surgery or chemotherapy. The presence of metastatic abdominal lymphnodes (until 3) did not excluded SRT. However, the indication to perform SRT was discussed in a multidisciplinary context. Notably, in few cases the transition from poly- to oligo-metastatic disease was obtained after chemotherapy; these patients were re-evaluated for SRT.

### SRT protocol

SRT was delivered according to a risk-adapted protocol; doses and fractionations were based on the size and location of the tumor (54 Gy/3 fractions, 55 Gy/5 fractions or 60 Gy/8 fractions). Treatment was delivered on alternate days regardless of the dose-fractionation regimen. A 4-D CT simulation scan was acquired for all patients. Respiratory gating was considered in cases where motion was > 7 mm in any direction. The gross tumor volume (GTV) was defined as the visible tumor on CT and PET imaging, and an internal GTV encompassed the GTV from all phases of respiration. A planning target volume (PTV) margin of 5 mm was used. The prescription point was approximately the 80% isodose line surrounding the PTV, with the requirement that 95% of the PTV was covered by 100% of the prescription dose. FDG-PET was performed before SRT and after 60 days from the treatment end.

### Statistical analyses and data presentation

Results of this study are predominantly descriptive and exploratory. Associations between responses to chemotherapy, SRT and clinical and pathologic variables (age, gender, oligo-metastatic disease, KRAS status, sites of disease) were evaluated by χ 2 test. P < 0.05 was considered statistically significant. Progression-free survival (PFS) was defined as the time elapsed from front-line treatment start to progression of the cancer as it occurred first; overall survival (OS) was defined as the time elapsed from the diagnosis to death from any cause. The Kaplan-Meier product limit method was applied to graph OS and PFS. Survival was measured from diagnosis in order to avoid generation of prognostic subgroups related to different treatments start times. Univariate analysis was done with the log-rank test. No attempt was done to perform multivariate analysis because of small number of cases. Statistical analysis was performed using the MedCalc^®^ 9.3.7.0 and Excel software.
